# The Boltzmann Equation and Its Place in the Edifice of Statistical Mechanics

**DOI:** 10.3390/e26121106

**Published:** 2024-12-18

**Authors:** Charlotte Werndl, Roman Frigg

**Affiliations:** 1 Department of Philosophy, Faculty of Social Sciences, University of Salzburg, Franziskanergasse 1, 5020 Salzburg, Austria; charlotte.werndl@plus.ac.at; 2 Department of Philosophy, Logic and Scientific Method, London School of Economics, Houghton Street, London WC2A 2AE, UK

**Keywords:** Boltzmann equation, Boltzmannian statistical mechanics, Gibbsian statistical mechanics, macro-state, BBGKY hierarchy

## Abstract

It is customary to classify approaches in statistical mechanics (SM) as belonging either to Boltzmanninan SM (BSM) or Gibbsian SM (GSM). It is, however, unclear how the Boltzmann equation (BE) fits into either of these approaches. To discuss the relation between BE and BSM, we first present a version of BSM that differs from standard presentation in that it uses local field variables to individuate macro-states, and we then show that BE is a special case of BSM *thus understood*. To discuss the relation between BE and GSM, we focus on the BBGKY hierarchy and note the version of the BE that follows from the hierarchy is “Gibbsian” only in the minimal sense that it operates with an invariant measure on the state space of the full system.

## 1. Introduction

In 1872, Boltzmann published one of his most influential papers, containing what is nowadays known as the Boltzmann equation (BE) ([1]; for a translation, see [2]). This paper was widely seen as a major breakthrough, and the BE has become a focal point of research, both formal and conceptual (see, for instance, [3] and references therein). Even though this research continues today, the Boltzmann equation occupies an uneasy position in the contemporary landscape. It is customary to classify approaches in statistical mechanics (SM) as belonging either to Boltzmanninan SM (BSM) or Gibbsian SM (GSM). BSM is today preferred by those working on the philosophy of SM, while GSM is the chosen approach among practitioners (for a synoptic discussion of these approaches, see [4] and references therein). Much can be said about this schism, and about the relation between BSM and GSM (see, for instance, [5,6]). But this is not our project in this paper. Here, we take the BSM-GSM schism for granted and ask how the BE relates to both BSM and GSM.

At first blush, the question whether the Boltzmann equation is Boltzmannian may sound absurd. “What else would it be? It’s the Boltzmann equation!”, one is tempted to respond. But the whiff of pleonasm quickly dispels once we recall that the monicker “BSM”, as used in contemporary discussions, is a neologism referring to a specific family of approaches going back to Boltzmann’s 1877 [7] paper. The formulation of what we now call BSM goes back to the Ehrenfests’ influential review [8]. Contemporary statements can be found, for instance, in [9,10,11]. So, in substance, the question is: how different are the approaches originating in the 1872 and 1877 papers, respectively? From the outset, the answer to this question was assumed to be “very different”, and the perception continues to the present day. In his 1877 paper [7], Boltzmann makes only passing reference to the BE in the context of reviewing the relation of probabilistic justifications of the second law. He then notes that he will now take a completely different path ([7], p. 166). This impression was reinforced in the Ehrenfests’ influential review [8], in which they discuss the *H*-theorem and the BE in the first chapter, entitled “The Older Formulation of Statistico-mechanical Investigations (Kineto-Statistics of the Molecule)”, just to then move on to discuss the 1877 approach in the second chapter, entitled “The Modern Formulation of Statistico-mechanical Investigations (Kineto-Statistics of the Gas Model)”. Contemporary versions of BSM follow in the Ehrenfests’ footsteps and offer “Boltzmannian” accounts of SM that stand squarely in the tradition of Boltzmann’s 1877 paper [7] and completely disregard the BE. Unsurprisingly, this vision on the relation between Boltzmann’s approaches is also reflected in the structure of reviews of the field, which treat them as distinct accounts of the subject matter and discuss them in separate sections (see, for instance, [4]). So, the situation we are faced with is one in which BSM and the BE are investigated side-by-side with little, if any, interaction between these investigations. But “separation in practice” does not mean that there are no points of contact or commonalities. But what these points of contact are, and where they might lie, is a largely unexplored question. Our aim in the first half of this paper is to fill this gap.

Turning to the other side of the schism, we ask how the BE relates to GSM. The latter originates in Gibbs’ seminal 1902 book [12], and, as noted, has become the go-to approach for the practitioner. Motivational concerns here pull in the opposite direction than with BSM because one might ask why anybody would think that these approaches would (or should) have anything in common. This reaction is correct as far as Boltzmann’s original derivation of the BE goes. Yet, even though GSM and the BE are prima facie rather different, points of contact have been noted. The so-called BBGKY hierarchy (which we discuss below) allows for the derivation of an equation that is formally equivalent to the Boltzmann equation. The fact that this derivation makes essential use of a probability density over the entire phase space of the system has led Uffink to note that “[t]he BBGKY approach is thoroughly Gibbsian in its outlook”, and to refer to the resulting equation as “[a]n ensemble-based analogy of the Boltzmann equation” ([4] p. 1037). This raises the question to what extent the derivation of the equation really is Gibbsian. The aim of the second part of this paper is to discuss this question.

The conclusion of our discussion is that the distance between the BE and BSM is much smaller than it appears to be at first sight. If a definition of macro-states can appeal to field configurations – as we argue it should – then states in BSM and the BE are defined in the same way; furthermore, their time-evolution turns out to be congruent at least for a finite timespan. The conclusion as regards the relation between the BE and GSM depends on how GSM is defined. On a minimal definition of GSM, the BE as derived in the BBGKY approach (which we refer to as the “marginal BE” for reasons that will become clear soon) is indeed Gibbsian; if a more demanding notion of GSM is adopted, then the situation is less clear.

This paper is structured as follows. We begin with a discussion of the essential ingredients of Boltzmann’s original derivation of the BE and the *H*-theorem (Section 2). We then turn to a discussion of the relation between the BE and BSM (Section 3). We pave the ground for this discussion by first introducing dynamical systems (Section 3.1) and then outline how macro-states in BSM can be defined by involving local field variables (Section 3.2). We proceed to generalising these variables and showing that the state description used in the BE fits the mould of generalised field variables as used in BSM (Section 3.3), and we review results showing that the dynamics of BSM and the BE are congruent for finite times (Section 3.4) and face the same exceptions (Section 3.5). We then change focus and turn to the BBGKY hierarchy and the derivation of the marginal BE in that approach (Section 4). We first discuss how this marginal BE relates to BSM (Section 5.1) and then introduce GSM (Section 5.2) to pave the ground for comparison of the BE with GSM (Section 5.3). We end with a conclusion (Section 6).

## 2. The Boltzmann Equation

We begin by retracing the main steps of Boltzmann’s original 1872 derivation of the BE (cf. [4,13]). Consider a gas and characterise its state by a time-dependent distribution function $f_{t}\left(\vec{v}\right)$, which gives us, at each point of time *t*, the relative number of molecules with a velocity between $\vec{v}$ and $\vec{v}+d^{3}\vec{v}$. Boltzmann’s aim in his paper was to formulate a time evolution for $f_{t}\left(\vec{v}\right)$ and then show that, in some sense, the distribution function approximates the continuous Maxwell–Boltzmann distribution.

In order to make progress on this, Boltzmann aimed to derive an equation that describes how the distribution function changes over time. To this end, he considered a monatomic gas with molecules of diameter *D* that behave like hard spheres and a container with perfectly elastic walls. He further had to make the assumptions that already in the initial state and at all later times each velocity direction is equally probable, and that at all times the relative number of molecules with their velocities in any given interval and their positions in a particular spatial region *R* does not depend on the location of *R* in the available volume (cf. [4]). He also assumed that the number of molecules is so large that the (discrete) distribution of their velocities can be well approximated by a continuous and differentiable function.

The next and strong assumption that Boltzmann made is the *Stosszahlansatz* (for a more detailed discussion of the Stosszahlansatz, see [3] and references therein). Now, use cylindrical coordinates and choose a frame of reference such that particle 1 with velocity $\vec{v_{1}}$ is at rest at the origin, and the relative velocity $\vec{v_{2}}-\vec{v_{1}}$ is directed along the negative *z* axis. Let (b,ϕ,z) denote the coordinates of the path of the centre of the second particle. It is then the case that *b* and ϕ are constant and $z\left(t\right)=z_{0}-∥\vec{v_{1}}-\vec{v_{2}}∥t$ before the collision. Note that when the particles are elastic hard spheres, a collision will take place only if the impact parameter *b* is less than the diameter *D* of the spheres. The Stosszahlansatz then states that the number $N(\vec{v_{1}},\vec{v_{2}})$ of collisions during a time dt in which the initial velocities $\vec{v_{1}}$ and $\vec{v_{2}}$ within an element $d^{3}\vec{v_{1}}d^{3}\vec{v_{2}}$ are changed into final velocities $\vec{v_{1}^{*}}$ and $\vec{v_{2}^{*}}$ within a spatial volume element $dV=bdbd\phi dz=∥\vec{v_{1}}-\vec{v_{2}}∥bdbd\phi dt$ is proportional to the product of the number of particles with velocity $\vec{v_{1}}$ within $d^{3}\vec{v_{1}}$ (i.e., $Nf\left(\vec{v_{1}}\right)d\vec{v_{1}}$), and those with velocity $\vec{v_{2}}$ within $d^{3}\vec{v_{2}}$ (i.e., $Nf\left(\vec{v_{2}}\right)d\vec{v_{2}}$), and that spatial volume element. That is,

(1) $$N\left(\vec{v_{1}},\vec{v_{2}}\right)=N^{2}f\left(\vec{v_{1}}\right)f\left(\vec{v_{2}}\right)∥\vec{v_{2}}-\vec{v_{1}}∥d^{3}\vec{v_{1}}d^{3}\vec{v_{2}}\;b\;db\;d\phi \;dt.$$

Because the collision laws are time-reversal invariant, a similar assumption is made about the so-called inverse collisions, where $\left(\vec{v_{1}^{*}},\vec{v_{2}^{*}}\right)$ are the initial velocities and $N(\vec{v_{1}},\vec{v_{2}})$ are the final velocities (in the past) (note that the collision laws entail that $∥\vec{v_{2}}-\vec{v_{1}}∥=∥\vec{v_{2}^{*}}-\vec{v_{1}^{*}}∥$ and $d^{3}\vec{v_{1}^{*}}d^{3}\vec{v_{2}^{*}}=d^{3}\vec{v_{1}}d^{3}\vec{v^{2}}$):

(2) $$N\left(\vec{v_{1}^{*}},\vec{v_{2}^{*}}\right)=N^{2}f\left(\vec{v_{1}^{*}}\right)f\left(\vec{v_{2}^{*}}\right)∥\vec{v_{2}^{*}}-\vec{v_{1}^{*}}∥d^{3}\vec{v_{1}^{*}}d^{3}\vec{v_{2}^{*}}\;bdbd\phi dt.$$

There is also an additional assumption (not stated in Boltzmann) that is needed to arrive at the Boltzmann equation, namely, that the density of the gas is so low that $f_{t}\left(\vec{v}\right)$ changes only under the effect of binary collisions (i.e., collisions of three-particles can be ignored) (cf. [4]). With all these assumptions in place, by taking the difference between Equations (1) and (2) and integrating over all variables except *t* and $\vec{v_{1}}$, Boltzmann derived a differentio-integral evolution equation for $f_{t}$, which is now known as the *Boltzmann equation*:

(3) $$\frac{\partial f_{t}\left(\vec{v_{1}}\right)}{\partial t}=N^{2}\int_{0}^{D}bdb\int_{0}^{2\pi }d\phi \int_{R^{3}}d^{3}\vec{v_{2}}∥\vec{v_{2}}-\vec{v_{1}}∥\left(f_{t}\left(\vec{v_{1}}\right)f_{t}\left(\vec{v_{2}}\right)-f_{t}\left(\vec{v_{1}^{*}}\right)f_{t}\left(\vec{v_{2}^{*}}\right)\right),$$

where the first two integrals are a trivial integration over a disk that yields $\pi D^{2}$.

In his 1872 paper, Boltzmann then went on to consider the quantity

(4) $$H\left[f_{t}\right]=\int f_{t}\left(\vec{v}\right)\ln\left(f_{t}\left(\vec{v}\right)\right)d^{3}\vec{v}.$$

Assuming that the Boltzmann equation holds for all times, he then showed that

(5) $$\frac{dH[f_{t}]}{dt}\leq 0,$$

a result which is now known as the *H*-theorem. Boltzmann furthermore showed that *H* is stationary just in case $f_{t}$ is the Maxwell–Boltzmann distribution, i.e.,

(6) $$\frac{dH[f_{t}]}{dt}=0\;\;\mathrm{if},\;\mathrm{and}\;\mathrm{only}\;\mathrm{if},\;\;f_{t}\left(v\right)=Ae^{-Bv^{2}},$$

where, of course, $v=|\vec{v}|$.

## 3. The Boltzmann Equation and BSM

We now ask how the Boltzmann equation relates to BSM. To pave the ground, we begin with a brief summary of BSM and dynamical systems. This is to introduce notation and to remind readers of the core concepts (for an extensive discussion of BSM, see [4] and references therein). We then discuss in some detail how macro-states in BSM are defined in non-equilibrium situations. This is important because out of equilibrium, macro-states are defined through field variables, and these connect naturally to the BE. A comparison between BSM and the BE then must focus on two issues: states and dynamics. As regards the former, the question is how the states of a system are described in both approaches and how the descriptions relate to each other. As regards the latter, we will ask how the two approaches describe the dynamics of a system and how these descriptions compare to each other.

### 3.1. Dynamical Systems and BSM

Statistical mechanics studies physical systems like gases in containers. Described mathematically, these systems have the structure of a *measure-preserving dynamical system*, i.e., a quadruple $\left(X,\Sigma_{X},\phi_{t},\mu \right)$. *X* is the state space of the system, i.e., a set containing all possible micro-states the system can be in. For a gas with *n* molecules, *X* has 6n dimensions: three dimensions for the position of each particle and three dimensions for the momentum of each particle. $\Sigma_{X}$ is a σ-algebra on *X* and μ is a measure on $\left(X,\Sigma_{X}\right)$, which is required to be invariant under the dynamics: $\mu_{X}\left(T_{t}\left(A\right)\right)=\mu_{X}\left(A\right)$ for all $A\in \Sigma_{X}$ and all *t*. The dynamics of the model is given by an *evolution function*$\phi_{t}:X\to X$, where t∈R if time is continuous and t∈Z if time is discrete. $\phi_{t}$ is assumed to be measurable in (t,x) and to satisfy the requirement $\phi_{t_{1}+t_{2}}\left(x\right)=\phi_{t_{2}}\left(\phi_{t_{1}}\left(x\right)\right)$ for all x∈X and all $t_{1},t_{2}\in R$ or Z. If, at a certain point of time $t_{0}$, the system is in micro-state $x_{0}$, then it will be in state $\phi_{t}\left(x_{0}\right)$ at a later time *t*. For systems that are governed by an equation of motion such as Hamiltons’s equation, $\phi_{t}$ corresponds to the solutions of this equation. The *trajectory* through a point *x* in *X* is the function $s_{x}:R\to X$, $s_{x}\left(t\right)=\phi_{t}\left(x\right)$ (and mutatatis mutandis for discrete time) (cf. [14]).

At the macro-level, the system is characterised by a set of macro-states, which describe the system in macroscopic terms. We will discuss how exactly macro-states ought to be defined in the next subsection. What matters from a conceptual point of view is that in BSM macro-states *supervene* on micro-states. A system’s micro-state therefore uniquely determines its macro-state. This determination relation is normally many-to-one. Therefore, every macro-state *M* is associated with a *macro-region* $X_{M}$ consisting of all micro-states for which the system is in *M*. A set of macro-states is complete if it contains all macro-states that the system can possibly be in. For a complete set of macro-states, the macro-regions form a partition of *X* (i.e., the different $X_{M}$ do not overlap and jointly cover *X*).

One of these macro-states is the equilibrium macro-state of the system. The macro-region corresponding to this macro-state is the largest of all macro-regions, and the dynamics of the system is such that if the system is initially prepared in a non-equilibrium macro-state, it approaches, and eventually reaches, the equilibrium macro-state, and, in the long run, spends most of the time there. Different versions of BSM disagree about why this is the case, and about how equilibrium should be defined (for a discussion of various approaches, see [6]). For the current discussion, these disagreements are immaterial because only the “end result” matters, and there is agreement on that.

### 3.2. Macrostates in BSM and Field Variables

We will now have a detailed look at how macro-states are defined in BSM. This is pivotal to the discussion because once we have the right understanding of macro-states, we see that there is a natural relation between how states are described in the BE and in BSM.

A common way to define macro-states is by appeal to Boltzmann’s original 1877 method [7], which is now commonly referred to as the “combinatorial argument”. Contemporary discussions of this argument can be found in [9] (Chapter 3) and [4] (Section 4.4). This argument provides important insights; most notably, it establishes the Maxwell–Boltzmann distribution as the equilibrium distribution of a dilute gas. At the same time, it faces technical limitations and conceptual issues that cast doubts on its suitability as general account of macro-states (for a discussion of these, see [4] (Section 4.4)).

To overcome these problems, we have proposed an alternative version of BSM [15]. Its key trait is that it takes “macro” in macro-states at face value and avoids appeal to micro-properties in the definition of macro-states. In thermodynamics, the state of a system is described by assigning values to macroscopic variables like volume, pressure, and temperature. The same idea should be applied in BSM. Hence, we submit that at the macro-level a system is characterised by a set of *lmacro-variables* (for some l∈N). From a mathematical point of view, macro-variables are measurable functions from *X* into another space V. That is, $v_{i}:X\to V_{i},x\to v_{i}\left(x\right)$, i=1,…,l. It is crucial for what follows that the $V_{i}$ can be different for different *i*. For some macro-variable, the space $V_{i}$ is R because the variables take values in the real numbers. This is the case, for instance, for internal energy and total magnetisation. But as we will see soon, this is generally not the case for variables like pressure and temperature, where the $V_{i}$ are function spaces.

With macro-variables in place, a *macro-state* is defined by the *values* that a set of macro-variables $\left\{v_{1},\ldots ,v_{l}\right\}$ assume. We adopt the notational convention that capital letters $V_{i}$ denote the values of variables $v_{i}$ and write $v_{i}\left(x\right)=V_{i}$ to express that variable $v_{i}$ assumes value $V_{i}$ when the system is in micro-state *x*. A macro-state is then defined by a particular set of values $\left\{V_{1},\ldots ,V_{l}\right\}$. That is, the system is in macro-state $M_{V_{1},\ldots ,V_{l}}$ iff $v_{1}=V_{1},\ldots ,v_{l}=V_{l}$. Note that in some cases exact values can turn out to be unsuitable to define macro-states and it is advisable to define them through intervals instead. Nothing in what follows depends on this.

We mentioned that for some variables, the space $V_{i}$ is R while for others, it is a function space. This is because in non-equilibrium situations, some physical variables do not assume the same values *everywhere* in the system. As an example, consider pressure. In equilibrium, the pressure of a gas is the same everywhere in the gas, and hence, one can describe the macro-state of the gas by saying what its pressure is. This is no longer possible when the system is out of equilibrium. The standard example with a gas approaching equilibrium after removing a partition wall illustrates this. A split second after the wall has been removed, there is no such thing "the" pressure of the gas. The pressure in the area originally occupied by the gas is still almost the same, while the pressure in the originally empty part is close to zero. Real-valued macro-variables cannot capture this situation. To describe situations like these, we have to regard pressure as a *field* that takes a value at every point in the container, and to describe the gas’ macro-state a split second after the removal of the wall, one has to specify the pressure field throughout the container.

The relevant difference in the examples above (and which is discussed in detail in [16]) is between *global variables*, which assign a real value to the entire system, and *local variables*, which assign a value to each point in ordinary physical space. Examples for the former are internal energy and total magnetisation; examples for the latter are pressure, temperature, and local magnetisation density. Local variables then require a description through field variables.

To introduce local field variables, we follow the convention of representing ordinary physical space by $R^{3}$. A *scalar field* on $R^{3}$ is a measurable function $f:R^{3}\to R$, $\vec{r}\to f\left(\vec{r}\right)$. That is, it is a measurable function that assigns to each point in space $\vec{r}$ a real number $f(\vec{r})$. If necessary, this definition can be restricted to a subset $S\in R^{3}$ and we can say that a scalar field on *S* is a measurable function f:S→R. We can now give a precise meaning to the notion of a local variable: to say that a quantity like pressure is “local” means that it is a scalar field $f:R^{3}\to R$.

To introduce macro-states, let us consider the set of all scalar fields on $R^{3}$ (or *S*). This set has the structure of a vector space. That is, the linear combination of any two scalar fields is again a scalar field, assuming the standard definition of the multiplication of functions with numbers and the addition of two functions. Let us denote this space by F. A particular assignment of values to each point of space is also called a *field configuration*. For example, $f(\vec{r})=3$ and $f\left(\vec{r}\right)=\left|\vec{r}\right|$ are field configurations. So, we can say that F is the space of field configurations. Recall that we said that there is no assumption that *all* $V_{i}$ in the definition of a macro-variable have to be R and that $V_{i}$ can in fact be any space. This point becomes important now because it allows us to take $V_{i}$ to be the space F, and doing so is the key to understanding local variables in BSM. Indeed, local variables are macro-variables for which $V_{i}$ is a space of scalar fields: they are macro-variables that assign to every point in space a scalar field. Accordingly, the “value” of a local variable $v_{i}$ is a field configuration. In sum, we can say that the relevant difference between global and local variables as introduced in the examples above is that global variables have the mathematical form of real-valued variables and local variables have the mathematical form of field-valued variables $f:R^{3}\to R$.

As we noted earlier, BSM in the version considered here defines macro-states through the values of a set $\left\{V_{1},\ldots ,V_{l}\right\}$ of values of the macro-variables $\left\{v_{1},\ldots ,v_{l}\right\}$: the system is in macro-state $M_{V_{1},\ldots ,V_{l}}$ iff $v_{1}=V_{1},\ldots ,v_{l}=V_{l}$. This remains the case, but with the qualification that if a macro-variable is a local variable, then its value is a field configuration. Macro-states are therefore individuated through values of macro-variables, as expected, and two macro-states are identical iff all variables assume the same values. It is obvious that the supervenience postulate bears out also when values can be field configurations: it is still the case that every macro-state *M* is associated with a *macro-region* $X_{M}$ consisting of all micro-states for which the system is in *M* and that for a complete set of macro-states, these macro-regions form a partition of *X*.

For what follows, it is important to note that we can generalise the local field variables just introduced in two ways. First, instead of using $R^{3}$ as the input space, we can consider field variables on $R^{n}$ where *n* is an arbitrary natural number. Second, we can free the input space from the interpretation as physical space and take it to be any space. This is obviously necessary if we work with $R^{n}$ for n≠3, but it is available as an option also for fields over $R^{3}$ because the formalism does not in any way depend on $R^{3}$ being interpreted as physical space. When generalising field variables in this way, the mathematical properties remain the same and such field variables are still macro-variables in the Boltzmannian sense discussed here. Yet, when stripped of an interpretation in terms of physical space, a field variable can no longer meaningfully be said to be “local”. For this reason, we reserve the term “*local* field variable” to cases where the relevant space is interpreted as physical space. The first generalisation is one we mention for the sake of completeness, and it will not play any role in what follows. By contrast, the second generalisation will be crucial in our discussion of the relation between the BE and BSM.

### 3.3. States

At first blush, the state descriptions offered by the BE and BSM seem to be rather different, in particular if one focusses on the standard version of BSM, which associates macro-states with a partitioning of the phase space without elaborating on how this partitioning is reached. The former then describes the state of a gas through a velocity distribution $f_{t}\left(\vec{v}\right)$, while the latter does so through macro-states that correspond to parts $X_{M}$ of the phase space *X* which jointly form a partition of *X*. But on closer inspection, the difference between the two frameworks turns out to be smaller than it appears. The BE describes non-equilibrium situations, and it shows how a distribution that is not initially in equilibrium eventually approaches equilibrium. This means that we have to work with a state description that is apt for non-equilibrium situations. As have seen in the previous subsection, in such situations the specification of a macro-states has to involve field variables. That is, the state is fixed by a set of measurable functions $v_{i}:X\to V_{i},x\to v_{i}\left(x\right)$, i=1,…,l, where the $V_{i}$ can be a space F of field configurations.

Now, this is precisely what happens in the BE if we allow for generalised field variables as introduced at the end of the previous section: the velocity distribution $f_{t}\left(\vec{v}\right)$ just *is* the field that assigns to each point in *X* the velocity distribution of the gas. More precisely, the velocity distribution $f_{t}\left(\vec{v}\right)$ is a function from $R^{3}\to R$, where $R^{3}$ represents the possible velocity coordinates of one particle. So, from a mathematical point of view, the velocity distribution in the BE has the same structure as a local field variable in BSM; the significant difference between them is that in the BE, $R^{3}$ is no longer interpreted as physical space, which makes it a generalized field variable as introduced at the end of the previous subsection. In fact, and this is the crucial point: from this point of view, the state description in the BE is of the same kind as the state description in BSM.

We note, however, that the distribution $f_{t}\left(\vec{v}\right)$ in the BE is a *velocity* distribution and, as such, it contains no information at all about the position of the particles. Hence, two initial conditions of the system *x* and *y* with the same initial velocity distribution, will, according to the BE, have the same $f_{t}\left(\vec{v}\right)$ and evolve in the same way even if the positions in *x* and *y* are different. In this sense, the BE offers only a partial state-description, while BSM has the resources to provide a full state description that contains information about both *velocity and position*. The fact that the BE omits position information is an important limiting factor. The standard examples of the approach to equilibrium is a gas that is confined to the left half of the container by a dividing wall. When the wall is removed, the gas is a non-equilibrium state and starts spreading until it fills the entire container evenly. The BE does not describe this process because it remains silent about the position of the gas particles. (We note that this limitation is specific to Boltzmann’s original approach. In Lanford’s approach position is considered).

In sum, far from there being a conflict between the state descriptions in the BE and BSM, the former is a special case of the latter.

### 3.4. Dynamics: Congruence

In BSM, the dynamics of the gas is given by the fundamental equations of motion, Hamilton’s equations. The BE, by contrast, is an equation describing the evolution of the velocity density $f(\vec{v})$, where the dynamics of the density emerges from assumptions about how molecules collide and no appeal to the fundamental Hamiltonian dynamics is made in the formulation of the BE. This raises the question of how the two time evolutions relate. An answer to this question comes from Lanford’s theorem.

Lanford [17,18,19,20] suggested tackling the problem of the relation between the two time evolutions by asking whether (and if so, in what sense) the Boltzmann equation is consistent with the underlying Hamiltonian dynamics. This way of thinking about the problem is based on a simple mathematical fact and a posit. The mathematical fact is that to every micro-state *x* corresponds a *unique* velocity distribution $f(\vec{v})$, which implies that the dynamics of *x* uniquely determines the dynamics of $f(\vec{v})$. The posit is that, fundamentally, a gas is a dynamical system whose micro-state evolves under the Hamiltonian equations of motion, which is true *irrespective* of how macro-states are described. This posit is, of course, in line with BSM, where the same assumption is made.

Lanford’s core idea then is to pick an initial condition *x* and do two things. First, consider the time evolution x(t) of this initial condition. Since a unique velocity distribution corresponds to every micro-state in x∈X, this is a fortiori true for all x(t) and so the time evolution of the initial condition naturally induces a time evolution of the velocity distribution $f_{x(t)}\left(\vec{v}\right)$. Second, take the velocity distribution corresponding to the initial condition *x* and use it as the initial distribution that is moved forward under the BE. Solving the BE for this initial distribution gives $f_{t}\left(\vec{v}\right)$. The question of the relation between the two dynamical laws then comes down to the question whether $f_{x(t)}\left(\vec{v}\right)$ and $f_{t}\left(\vec{v}\right)$ are consistent.

At this point, a technical issue emerges. For a gas with a finite number of particles, the *exact* velocity distribution F[x,n] of a state *x* is a sum of delta functions. Such a distribution is neither continuous nor differentiable, and hence, the BE is not able to say how it evolves over time. To get around this problem, Lanford considers the exact distribution for a very large number of particles and then asks whether the following conditional is true: if F[x,n] is close to the continuous density $f_{x(t)}\left(\vec{v}\right)$, then the later distribution of state $F[x_{t},n]$ is close to $f_{t}\left(\vec{v}\right)$, where $f_{t}$ is the solution of the Boltzmann equation with initial state $f_{0}\left(\vec{v}\right)$. With some further technical assumptions in place, including a measure-theoretic analogue of the Stosszahlansatz (cf. [3,4]), Lanford arrived at the following theorem, now known as Lanford’s theorem: *As judged from the microcanonical measure on phase space restricted to states x that have their exact distribution of state F[x,n] close to a continuous function $f_{0}\left(\vec{r},\vec{v}\right)$, a very large proportion of initial states evolve under the Hamiltonian dynamics in such a way that their later exact distribution of state $F[x_{t},n]$ is close to the function $f_{t}\left(\vec{r},\vec{v}\right)$, which results from evolving $f_{0}\left(\vec{r},\vec{v}\right)$ under the Boltzmann equation, whenever $t<t_{*}$ for a certain amount of time $t_{*}$*. As always, “a very large portion” means a set of states of measure ε for ε<<1. This implies that $f_{x(t)}\left(\vec{v}\right)$ is consistent with $f_{t}\left(\vec{v}\right)$ for $t<t_{*}$.

Lanford’s theorem is a remarkable achievement as it shows that the BE, which was derived solely based on assumptions about particle collisions, is actually consistent with the underlying fundamental dynamics. It is, however, important to bear its assumptions and limitations in mind. The theorem depends on a measure-theoretic version of the Stosszahlansatz (cf. [3,4]), and the result can only be shown for extremely rarified gases; more specifically, the proof relies on the so-called Bolzmann–Grad limit. The Boltzmann–Grad limit is a mathematical limiting procedure through which, under a specific set of assumptions, a continuous distribution is obtained from the discrete distribution of state $F[x_{t},n]$ as *n* goes to infinity. Two major assumptions involved are that the gas is so dilute that collisions involving three or more particles are so rare that they can be ignored, but that the gas is not so dilute that collisions do not make a contribution to the evolution of the distribution function at all (see [4] (Section 6.5)). But even if one were to regard these as unproblematic (or at least justifiable), there are two further restrictions that cut to the heart of the matter. These are that the distributions can be proven to be consistent only for $t<t_{*}$, and that this is the case only for a very large portion of initial conditions. We discuss the former now and postpone a discussion of the latter to the next subsection.

It can be shown by order of magnitude considerations that $t_{*}$ is roughly 40% of the mean collision time (i.e., the duration between collisions). Unfortunately, this is a very short period of time: in air at room temperature and normal atmospheric pressure, this is in the order of microseconds. Consequently, the theorem does not help in justifying the usual applications of the Boltzmann equation to macroscopic phenomena which demand a much longer time-scale. Nevertheless, the time-scale is not trivially short: in two fifths of the mean duration between collisions, approx. 40 percent of particles will have been involved in a collision.

It is important to be careful about the logic of the theorem. The theorem makes the positive statement that for $t<t_{*}$, the dynamics of the BE is consistent with the underlying Hamiltonian dynamics. It does *not* make the converse negative statement that this is not the case for $t>t_{*}$; the theorem is simply silent about what happens after $t_{*}$. It does not preclude that the two dynamics are consistent for much longer; it simply does not assert that this is the case. This raises the question as to the reasons of the theorem’s limitation. The complexities of this issue are not yet fully understood, and there are open questions. However, we do expect consistency to break down at some point. As we will see in the next subsection, the exact Hamiltonian dynamics exhibits time-reversal invariance and Poincaré recurrence. By contrast, the dynamics of the BE implies a monotonic decrease of $H[f_{t}]$, which is *incompatible* with both of these features. So, the dynamics of the BE differs from the underlying Hamilton dynamics and hence cannot be universally valid.

### 3.5. Dynamics: Time Reversal and Exceptional States

In his 1872 paper, Boltzmann seemed to believe that the assumptions in his derivations such as the Stosszahlansatz were exceptionless. Hence, he also seemed to believed that *H* always decreases monotonically and that, with his *H*-theorem, he had derived a rigorous, analytical, and general proof of the Second Law of Thermodynamics (cf. [4] (Section 4.2); [21] p. 73). We note that there is some debate about this point. Van Plato ([22] (p. 81)), for instance, takes the opposite view and argues that, already in his 1872 paper, Boltzmann was well aware that his theorem had exceptions and did not believe that he had derived the Second Law. Whatever one’s views on Boltzmann’s position in 1872, a revision of the universal validity of the Boltzmann equation and the *H*-theorem became unavoidable in the light of two features of the underlying dynamics. First, the dynamics is time-reversal invariant. This is what drives Loschmidt’s [23] reversibility objection, which states that if a system is allowed to evolve from a state of high *H* to a state of low *H*, the reverse trajectory from low *H* to high *H* is also allowed. Second, the dynamics exhibits Poincaré recurrence. This is what drives Zermelo’s [24,25] recurrence objection, stating that a classical system can be shown to return arbitrarily close to its initial state when enough time has passed, which implies that transitions from low values of *H* to high values of *H* happen if we wait long enough. These objections imply that states with lower values of *H* can evolve to states with higher values of *H*, contradicting the universality of the H-theorem. There is some debate about how exactly Boltzmann revised his initial views in his [7,26,27] in the light of the reversibility and recurrence objections (see [4] (Sections 4.3 and 4.5)) for a discussion and [3] for an in-depth discussion of time-reversal in the BE). But whatever the precise trajectory of Boltzmann’s thinking on this may have been, he eventually seems to have converged to the view that was later called the statistical interpretation of the BE: the BE is valid most of the time and hence the actual evolution of the distribution function stays close to the solution of the BE most of the time, but there are exceptional times when this is not the case. He may also have thought, although that is less clear, that there may be exceptional initial states that do not exhibit a uniform decrease of *H* (and hence, convergence toward the Maxwell–Boltzmann distribution), but that these states were such that they could rarely, if ever, be seen in nature.

It pays noting that this is compatible with Lanford’s approach, although the emphasis in the latter is different: Lanford derives a result that holds for all times smaller than a certain cut-off time and for a large number of (but not all) initial conditions. But, as we have seen, this time is very short and Lanford’s theorem remains silent about what happens for times larger than the cut-off time. It is entirely conceivable that Boltzmann’s exceptional times when the system does not evolve according the BE occur for times larger than the cut-off. Furthermore, Lanford explicitly excludes some initial states of measure α, which is argued to be small. This is in fact Boltzmann’s idea that there are exceptional states.

The conclusion is that the BE in the statistical interpretation is compatible with BSM, which incorporates time reversal invariance and Poincaré recurrence, because it allows for moments of increasing *H*, which can be due either to the BE not being valid for all times or because the system starts off in a very special initial condition (different versions of BSM do this in different ways and for a discussion, see [6] (Chapter 3)). This also means that the BE is compatible with thermodynamic-like behaviour, whereby the evolution of the system is such that it spends most of the time close to equilibrium, from which it exhibits frequent small fluctuations and rarer large fluctuations [5].

### 3.6. Summing Up

The conclusion of our discussion is that first appearances notwithstanding, the BE and BSM are closely related. The state description in the BE is a special case of the state description in BSM, provided that states in BSM are described by dint of scalar fields (as we argue they should). The dynamics of the BE is different from the underlying Hamiltonian dynamics, but at least under a statistical interpretation, the BE allows for time-reversal invariance and Poincaré recurrence, and it can exhibit thermodynamic-like behaviour.

## 4. The BBGKY Hierarchy and the Marginal Boltzmann Equation

The BBGKY hierarchy (named after Bogoliubov, Born, Green, Kirkwood, and Yvon) is a set of hierarchically structured equations that describe the evolution of large systems by dint of marginal probability densities. This approach is relevant in the current context because the first of these equations is formally equivalent to the Boltzmann equation, and for reasons that will become clear soon we dub it the “Marginal Boltzmann Equation” (MBE). To pave the ground for a discussion of the MBE, we now briefly summarise the main tenets of the BBGKY hierarchy. The presentation of the BBGKY approach in this paper follows [4] (Section 6.5).

Consider a system of particles with states $x=(\vec{q_{1}},\ldots ,\vec{q_{n}},{\vec{p}}_{1},\ldots {\vec{p}}_{n}$), n∈N where the $\vec{q_{i}}$ and $\vec{p_{i}}$ are the position and momentum coordinates of the *i*-th particles. For relevant systems in statistical mechanics, the Hamiltonian will be symmetric under the permutation of the particles. The BBGKY approach restricts attention to symmetric Hamiltonians, and it assumes that these contain no terms that depend on three or more particles. These assumptions are physically motivated and systems of interest typically meet them. A Hamiltonian for a system of *n* indistinguishable particles is a case in point:

(7) $$H\left({\vec{q}}_{1},\ldots ,{\vec{q}}_{n},{\vec{p}}_{1},\ldots ,\vec{p_{n}}\right)=\sum_{i=1}^{n}\frac{{\vec{p_{i}}}^{2}}{2m}+\sum_{i=1}^{n}V\left({\vec{q}}_{i}\right)+\sum_{i<j}^{n}\phi (||{\vec{q}}_{i}-{\vec{q}}_{j}||),$$

where ϕ is the interaction potential between particle *i* and particle *j*, and *V* is the potential that represents the walls of the bounded spatial region Λ:

(8) $$V\left(\vec{q}\right)=0\;\;for\;\;\vec{q}\in \Lambda ;\;\;\mathrm{and}\;\;\infty \;\;\mathrm{otherwise}.$$

Next, consider an arbitrary time-dependent probability density $\rho_{t}\left(x\right)$ on state space *X*. The Liouville-equation determines the evolution of $\rho_{t}$ governed by the Hamiltonian *H*:

(9) $$\frac{\partial \rho_{t}}{\partial t}=\sum_{i=1}^{n}\frac{\partial H}{\partial {\vec{p}}_{i}}\frac{\partial \rho_{t}}{\partial {\vec{q}}_{i}}-\frac{\partial H}{\partial {\vec{q}}_{i}}\frac{\partial \rho_{t}}{\partial {\vec{p}}_{i}}.$$

We now define a sequence of marginal probability density functions of $\rho_{t}$ (note that ${\vec{x}}_{i}=\left({\vec{q}}_{i},{\vec{p}}_{i}\right)$):

(10) $$\rho_{t}^{\left(1\right)}\left({\vec{x}}_{1}\right)=\int \rho_{t}\left(x\right)d{\vec{x}}_{2}d{\vec{x}}_{3}\ldots d{\vec{x}}_{n},$$

and, more generally, for 1≤i≤n−1

(11) $$\rho_{t}^{\left(i\right)}\left({\vec{x}}_{1},\ldots {\vec{x}}_{i}\right)=\int \rho_{t}\left(x\right)d{\vec{x}}_{i+1}\ldots d{\vec{x}}_{n}.$$

In general, if we consider a probability density f(y,z) and then calculate the marginal by integrating over *z*, then the marginal can be interpreted as the probability of *y* given a particular value of *z* if one then averages over *z*. This is often paraphrased as the marginal specifying of the probability of *y*, while *z* may assume any value. Accordingly, $\rho_{t}^{m}$ is the probability density that particles 1,…,m take specific positions ${\vec{q}}_{1},\ldots {\vec{q}}_{m}$ and momenta ${\vec{p}}_{1},\ldots {\vec{p}}_{m}$ when the positions and momenta of the other particles have been averaged over, or all remaining particles occupy arbitrary positions and momenta.

As noted previously, we consider Hamiltonians that are symmetric under the permutation of the particles. The symmetry of the Hamiltonian per se does not imply anything about the symmetry of $\rho_{t}$. Yet, given that the permutation symmetry of the Hamiltonian reflects a physical property of the system, it makes sense to require that $\rho_{t}$ has the same symmetry. Under this assumption $\rho_{t}^{m}$ specifies the probability density for any arbitrarily chosen set of *m* particles to take specific positions $q_{1},\ldots q_{m}$ and momenta $p_{1},\ldots p_{m}$, whereas all remaining particles occupy arbitrary positions and momenta.

The guiding idea of the BBGKY approach is that instead of considering the time evolution of $\rho_{t}$, we can focus on the hierarchy of marginals defined through Equation (11). A particular case is the evolution of the one-particle marginal probability distribution $\rho_{t}^{\left(1\right)}$ (10). The question then is how these marginals evolve over time. It is not difficult to see that for the Hamiltonian (7), the evolution of $\rho_{t}^{\left(1\right)}$ is given by (cf. [4] (Section 6.5)):

(12) $$\frac{\partial \rho_{t}^{\left(1\right)}\left({\vec{x}}_{1}\right)}{\partial t}=L_{1}^{\left(1\right)}\rho_{t}^{\left(1\right)}\left({\vec{x}}_{1}\right)+\int d{\vec{x}}_{2}L_{1,2}^{\left(2\right)}\rho^{\left(2\right)}\left({\vec{x}}_{1},{\vec{x}}_{2}\right);$$

and, more generally, for higher-order reduced distribution functions, $\rho_{t}^{\left(i\right)}\left({\vec{x}}_{1},\ldots {\vec{x}}_{i}\right)$, the evolution is given by

(13) $$\begin{array}{ccc} & & \frac{\partial \rho_{t}^{\left(i\right)}\left({\vec{x}}_{1},\ldots ,{\vec{x}}_{i}\right)}{\partial t}=\sum_{j=1}^{i}L_{j}^{\left(1\right)}\rho_{t}^{\left(i\right)}\left({\vec{x}}_{1},\ldots ,{\vec{x}}_{i}\right)+\\ & & \sum_{j,r;j\leq r;1\leq j<i}L_{j,r}^{\left(2\right)}\rho^{\left(i\right)}\left({\vec{x}}_{1},\ldots ,{\vec{x}}_{i+1}\right)+\sum_{j=1}^{i}\int d{\vec{x}}_{i+1}L_{j,i+1}^{\left(2\right)}\rho^{\left(i+1\right)}\left({\vec{x}}_{1},\ldots ,{\vec{x}}_{i+1}\right), \end{array}$$

where

(14) $$L_{j}^{\left(1\right)}:={\vec{p}}_{j}\frac{\partial }{\partial {\vec{q}}_{j}}$$

and

(15) $$L_{j,r}^{\left(2\right)}:=\frac{\partial \phi (||q_{j}-q_{r}||)}{\partial {\vec{q}}_{j}}\left(\frac{\partial }{\partial {\vec{p}}_{j}}-\frac{\partial }{\partial {\vec{q}}_{j}}\right).$$

Equations (12) and (13) define the BBGKY hierarchy, and we refer to these equations as the “BBGKY equations”. The hierarchy is equivalent to the Hamiltonian equation of motion under the above assumptions (i.e., that both *H* and $\rho_{t}$ are symmetric under permutations of the particle labels, and that *H* contains no terms that depend on three or more particles). It is no surprise that solving the equations of the BBGKY hierarchy turns out to be as difficult as solving the original Hamiltonian equations of motion. The formal manifestation of this difficulty is that the BBGKY equations are not closed: if one wants to know how $\rho_{t}^{\left(1\right)}$ evolves, one needs to know how $\rho_{t}^{\left(2\right)}$ evolves; if one wants to know how $\rho_{t}^{\left(2\right)}$ evolves, one needs to know $\rho_{t}^{\left(3\right)}$; and so on.

The main reason why the BBGKY hierarchy is interesting in the current context is that one can solve the equations if one cuts off the hierarchy, i.e., if one assumes for some finite *i* that $\rho^{\left(i\right)}$ is a functional of $\rho^{\left(r\right)}$ with r<i. In physical terms, this means that the correlation between *i* particles can be expressed in terms of correlations between fewer particles or the probability of finding one particle in a specific region of the one-particle space. In particular, if one considers the simplest case for i=2, a possible assumption is that $\rho^{\left(2\right)}$ factorizes in the distant past (i.e., t→−∞), yielding the following:

(16) $$\lim_{t\to -\infty }\rho_{t}^{\left(2\right)}\left({\vec{x}}_{1},{\vec{x}}_{2}\right)=\rho_{t}^{\left(1\right)}\left({\vec{x}}_{1}\right)\rho_{t}^{\left(1\right)}\left({\vec{x}}_{2}\right).$$

In essence, this assumption amounts to requiring that the molecular states of any pair of particles are uncorrelated before their interaction. This is analogous to the Stosszahlansatz (1), but formulated differently in terms of the marginal distribution functions of an ensemble.

Now, consider the homogeneous case, i.e., when $\rho^{\left(2\right)}$ is uniform over the position coordinates, i.e., when $\rho^{\left(2\right)}\left({\vec{x}}_{1},{\vec{x}}_{2}\right)$ can be expressed as $\rho^{\left(2\right)}\left({\vec{v}}_{1},{\vec{v}}_{2}\right)$. Then, it can be shown that when ϕ is an interaction potential of finite range and Equation (16) is assumed, Equation (12) is equivalent to the following:

(17) $$\frac{\partial \rho_{t}^{\left(1\right)}\left({\vec{v}}_{1}\right)}{\partial t}=N^{2}\int_{0}^{D}bdb\int_{0}^{2\pi }d\phi \int_{R^{3}}d^{3}\vec{v_{2}}∥\vec{v_{2}}-\vec{v_{1}}∥\left(\rho_{t}^{\left(1\right)}\left(\vec{v_{1}}\right)\rho_{t}^{\left(1\right)}\left(\vec{v_{2}}\right)-\rho_{t}^{\left(1\right)}\left(\vec{v_{1}^{*}}\right)\rho_{t}^{\left(1\right)}\left(\vec{v_{2}^{*}}\right)\right).$$

This equation is formally equivalent to the BE, with the important difference that the density in it is not the distribution function $f_{t}\left(\vec{v}\right)$, which specifies the relative number of molecules with a velocity between $\vec{v}$ and $\vec{v}+d^{3}\vec{v}$, but the first marginal of the probability density $\rho_{t}\left(x\right)$ on state space *X*. For this reason, we refer to this equation as the “Marginal Boltzmann Equation”. In other words, what the BBGKY derivation shows is that under the assumptions stated above, the rate of change of the marginal density describing the probability of one particle being in a certain state is *also* given by the Boltzmann equation.

## 5. The Marginal Boltzmann Equation, BSM, and GSM

We now ask how the MBE relates to BSM on the one hand, and to GSM on the other hand. We first deal with BSM, and then turn to GSM. To aid the discussion of the latter, we begin with a summary of GSM. As above, this is mainly to introduce notation and to remind readers of the core concepts.

### 5.1. The MBE and BSM

How, if at all, does the MBE fit into BSM? As above, it is helpful to first discuss how states are described and then turn to the dynamics. Since we now understand the relation between the BE and BSM, the strategy throughout is to compare the MBE to the BE; in as far as the MBE aligns with BE, it also aligns with BSM, because, as we have seen in the previous section, the BE is aligned with BSM. So, we explore this avenue first. If that fails, then there is, in principle, still the option that there is a connection between the MBE and BSM that “bypasses” BE. However, as we will see, while this is a conceptual possibility, it is not one that bears fruits.

Let us begin with states. As emphasised in the previous section, the MBE is an equation about the one-particle marginal probability density: the density describing the probability of finding one particle in a specific velocity range when averaging over the velocities of all other particles. By contrast, the density in the BE is an actual frequency distribution of a specific state *x* of a gas. The MBE is not about such a distribution; the MBE specifies the probability of finding *one particle* in a specific region of one-particle space when repeatedly looking at that particle. For this reason, the MBE is not about the evolution of the actual distribution corresponding to a micro-state *x* as the system evolves over time, as in Boltzmann’s and Lanford’s approaches. And this is not merely an interpretational difference that one could set aside as a philosopher’s concern that is irrelevant to practitioners. In fact, one can prove that the one particle distribution of the MBE is the expected value (when computed relative to the distribution $\rho_{t}$) of the frequency distribution in the BE ([4] p. 1037):

(18) $$\langle f_{t}\left(\vec{v}\right)\rangle =\rho_{t}^{\left(1\right)}\left({\vec{v}}_{1}\right),$$

which goes to show that the densities in the MBE and in the BE offer genuinely different descriptions of the state of a gas. Furthermore, as [4] (ibid.) notes, the BE offers a more informative description of the system’s state because it describes the time evolution of the exact distribution of state *x*, while the MBE only describes the time evolution of the *average* of that exact distribution. Since, as we have seen, the state description in the BE is closely aligned with BSM, the state description in the MBE does not have the same connection to BSM as BE.

This, as noted, leaves open the possibility that the MBE has a connection to BSM that is independent of, and different from, BE’s connection to BSM. However, it is difficult to see what this connection would be. BSM does not consider one-particle marginal probability densities, and the procedures in BSM do not concern how probability densities such as the one-particle marginal distribution evolve forward in time. To be sure, there is nothing in BSM that precludes considering a one-particle marginal distribution and asking how it evolves over time, but this is something that is entirely foreign to BSM and hence fails to connect to its practices and methods.

Let us then turn to the dynamics. As noted previously, the left-hand side of the MBE is interpreted entirely differently than in the BE: it is interpreted as the one-particle marginal probability distribution in contrast to the relative number of molecules with a certain velocity. This has implications for the assessment of the congruence of the equation with the underlying Hamiltonian dynamics. Lanford’s theorem now no longer provides the formal underpinning that it provides for Boltzmann’s original derivation. Lanford’s theorem shows that, for a very large proportion of initial conditions, the distribution function obtained by solving the Boltzmann equation for time *t* given the initial velocity distribution of initial state *x* leads to the same result as first evolving *x* forward under the Hamiltonian equations until time *t* and then considering the velocity distribution given by the evolved state. Yet, considering the velocity distribution of the evolved state is of no relevance if what is considered is the one-particle marginal probability distribution.

At this point, we want to draw attention to a question, which, as far as we can tell, is not discussed in the extant literature. As we have seen above, in response to problems with time-reversal invariance and Poincaré recurrence, the BE is given a statistical interpretation, which effectively limits its applicability. As far as we are aware, no such restrictions have been formulated for the MBE, which would suggest that it is regarded as universally valid. There is a question whether this is sustainable. Time-reversal invariance and Poincaré recurrence are general features of the underlying dynamics, but since the first marginal is about an expectation value, it might be the case that its evolution is not in conflict with the said features of the underlying dynamics. This is a point that deserves further attention.

### 5.2. Gibbsian Statistical Mechanics

To pave the ground for our discussion of the relation between the MBE and GSM, we now provide a short summary of GSM (for an extensive discussion of GSM see, for instance, [4,28], and references therein).

The core object that is studied in GSM is a probability density (or distribution) ρ(x,t) over *X*. In Gibbs’ 1902 book [12] original presentation, ρ(x) is described as representing an ensemble, an infinite collection of independent systems that are governed by the same laws of motion but are in different initial states. There are alternative presentations that endeavour to avoid reference to ensembles; they regard GSM simply as a probabilistic algorithm. What follows does not depend on these interpretational issues and, hence, we set this question aside. Different interpretations of GSM are discussed in [29]. The density ρ(x,t) describes the probability of finding the state of a system in a region R⊆X at time *t*:

(19) $$p_{t}\left(R\right)=\int_{R}\rho \left(x,t\right)dx.$$

On physical grounds, the probability density must be conserved, meaning that for every region R(t) of *X* that is moving forward under the time evolution $\phi_{t}$, the probability must be constant. If the time evolution is generated by Hamiltonian equations of motion, this is the case if, and only if, Liouville’s equation holds.

Gibbs ([12], p. 8) introduces what he refers to as the *condition of statistical equilibrium*: a probability density that is in statistical equilibrium has to be stationary. That is, it does not change under the dynamics of the system: ρ(x,t)=ρ(x) for all *t*. There are usually a large number of stationary density functions for a given $\phi_{t}$. Hence, the question arises which of these is best to characterise a given physical situation. According to Gibbs, the so-called *microcanonical distribution* describes the equilibrium of a physical system which is completely isolated from its environment. It is the constant distribution on the system’s energy hypersurface H(x)=E. The *canonical distribution* should be used when the system is in contact with a heat bath. It is given by $e^{-H(x)/kT}/\zeta_{T}$, where *H* is the system’s Hamiltonian, *T* is the temperature, *k* is the Boltzmann constant, and $\zeta_{T}$ is the so-called *partition function* (for a discussion how to justify the choice of these distributions, see [30]).

How do Gibbsian probability densities connect to observations on physical systems? That is, what does an experimentalist observe when measuring, say, the magnetisation of a sample of iron? To reply to this question, first note that at the macro-level, a system is characterised by a set of *macro-variables*, associating a value with each point in state space *X*. Examples of macro-variables include volume, internal energy, and magnetisation. Mathematically speaking, macro-variables are measurable real-valued functions on the state space, i.e., g:X→R. For example, if *f* is the magnetisation of the system and the system is in micro-state *x*, then f(x) is the magnetisation of the system.

One can then introduce the *phase average* 〈f〉 of a macro-variable *f*:

(20) $$\langle f\rangle =\int_{X}f\left(x\right)\rho \left(x,t\right)dx.$$

When the system is in statistical equilibrium, it follows that 〈f〉 is time-independent. Standardly, a connection between the Gibbsian probability density and observable results is established by appealing to the *averaging principle* (AP). According to this principle, when observing the physical quantity associated with *f* on a system in equilibrium, the observed equilibrium value of *f* is the phase average 〈f〉. The principle is common and stated in many textbooks. For example, Chandler calls AP “[t]he primary assumption of statistical mechanics” ([31] p. 58), and Pathria and Beale state that they regard AP as the “the most important result” in SM ([32] p. 31). However, the status and scope of AP is a contested matter and there are alternative interpretations of GSM that do not accept AP (for instance, Wallace in [33] is sceptical about AP, and textbooks that do not explicitly endorse AP include [34,35]). However, even on such interpretations phase averaging is part of the practice of GSM and so calculating averages is, in one way or other, an essential part of GSM no matter how the theory is interpreted, and this is all that matters for the current discussion.

### 5.3. The MBE and GSM

As mentioned in the Introduction, the BBGKY hierarchy, and with it the MBE, have been seen as closely associated with GSM. Uffink is explicit when he says that the “BBGKY approach is thoroughly Gibbsian in its outlook, i.e., it takes a probability density over phase space as its basic conceptual tool […] An ensemble-based analogy of the Boltzmann equation comes out of this approach as a first-order approximation for dilute gases with collision times much smaller than the mean free time” ([4] p. 1037). In a similar vein, Wallace discusses the “necessity of Gibbsian statistical mechanics”, which he associates with explicitly probabilistic methods, and then notes “even for Boltzmann-apt systems, there are important cases where probabilistic methods seem necessary and do not reduce to Boltzmannian methods in any simple way. (Multi-time correlation functions by no means exhaust the list of such cases—indeed, I discuss another, the modern (‘BBGKY’) derivation of Boltzmann-type equations through truncation of the *N*-particle probability distribution to 2-particle marginals, in Wallace (2016)—but they already suffice to make the point)” ([36], (pp. 594–595)).

Whether the the MBE is “Gibbsian” to large extent depends on what is meant by “Gibbsian”. If considering a probability density over the entire state space is all it takes to be Gibbsian, then the MBE is indeed Gibbsian. However, we note that this is a rather weak notion of something being Gibbsian for two reasons. First, considering probability densities over a state space is common practice in many areas of science that have no obvious connection to GSM; in particular, it is deployed in areas other than physics, such as in mathematical population dynamics (e.g., the logistic equation; see [37,38]) or in modelling in economics (e.g., [37]). It is also common practice in ergodic theory (when the invariant measure is considered; see [14]), in Hamiltonian mechanics (for instance, when considering Liouville’s theorem; see [34]), in non-linear dynamics (for instance, when a dynamic is classified as dissipative because a volume element shrinks over time; see [37]), and, indeed, in BSM when the relative sizes of macro-regions are compared. All these would then have to count as Gibbsian, too. Second, other elements of GSM play no role in the BBGKY approach. Among these elements are the three ensembles central to GSM (micro-canonical, canonical, and grand-canonical), and an understanding of equilibrium in terms of stationarity and the averaging principle, which associates observable results with phase averages under certain circumstances (there is no agreement about what these circumstances are—see [29]). For sure, there is no conflict between any of these and the BBGKY approach, and there is potential for cross-fertilization; indeed, Uffink mentions that the BBGKY approach “gives an enormous extension of Gibbs’ own work by providing a systematic hierarchy of evolution equations for reduced (or marginalized) density functions, which can then be subjected to the techniques of perturbation theory” ([4] (ibid.)). One can only agree with this, but the question remains whether providing such an extension warrants the qualification of the approach itself as “Gibbsian”.

## 6. Conclusions

We have argued that the difference between the BE and BSM is much smaller than it seems at first sight. If the characterisation of macro-states can involve field configurations, then states in BSM and the BE are defined in the same way. Additionally, the time evolutions in the BE and BSM turns out to be congruent at least for a finite timespan. The situation as regards the relation between the BE and GSM depends on how GSM is defined. The BE as derived in the BBGKY approach is indeed “Gibbsian” if we assume a minimal definition of that term; the situation is, however, less clear if a more demanding notion of GSM is adopted.

## Data Availability

Data is contained within the article.

## Abbreviations

The following abbreviations are used in this manuscript:

BSM	Boltzmannian Statistical Mechanics
GSM	Gibbsian Statistical Mechanics
SM	Statistical Mechanics
BE	Boltzmann Equation
MBE	Marginal Boltzmann Equation
